# Diphtheria toxin‐based anti‐human CD19 immunotoxin for targeting human CD19^+^ tumors

**DOI:** 10.1002/1878-0261.12056

**Published:** 2017-04-04

**Authors:** Qian Zheng, Zhaohui Wang, Huiping Zhang, Qi Huang, Joren C. Madsen, David H. Sachs, Christene A. Huang, Zhirui Wang

**Affiliations:** ^1^ Center for Transplantation Sciences Massachusetts General Hospital and Harvard Medical School Boston MA USA; ^2^ TBRC Laboratories Center for Transplantation Sciences Massachusetts General Hospital and Harvard Medical School Boston MA USA; ^3^ Division of Cardiac Surgery Massachusetts General Hospital and Harvard Medical School Boston MA USA

**Keywords:** CD19^+^ tumor, diphtheria toxin, human CD19, immunotoxin, *Pichia Pastoris*

## Abstract

CD19 is expressed on normal and neoplastic B cells and is a promising target for immunotherapy. However, there is still an unmet need to further develop novel therapeutic drugs for the treatment of the refractory/relapsing human CD19^+^ tumors. We have developed a diphtheria toxin‐based anti‐human CD19 immunotoxin for targeting human CD19^+^ tumors. We have constructed three isoforms of the CD19 immunotoxin: monovalent, bivalent, and foldback diabody. *In vitro* binding affinity and efficacy analysis demonstrated that the bivalent isoform had the highest binding affinity and *in vitro* efficacy. The *in vivo* efficacy of the CD19 immunotoxins was assessed using human CD19^+^ JeKo‐1 tumor‐bearing NOD/SCID IL‐2 receptor γ^−/−^ (*NSG*) mouse model. In these animals, CD19 immunotoxins significantly prolonged the median survival from 31 days in controls to 34, 36, and 40 days in animals receiving the monovalent isoform, foldback diabody isoform, and bivalent isoform, respectively. The bivalent CD19 immunotoxin is a promising therapeutic drug candidate for targeting relapsing/refractory human CD19^+^ tumors.

**Abbreviations**
7‐AAD: 7‐aminoactinomycin D
ATP: adenosine triphosphate
B‐ALL: B‐cell acute lymphoblastic leukemia
BID: bis in die
BiscFv: bivalent single‐chain variable fragment
CLL: chronic lymphatic lymphoma
CSC: China Scholarship Council
DT: diphtheria toxin
G_4_S: four glycine residues and one serine residue
His: histidine
IACUC: Institutional Animal Care and Use Committee
IC_50_: half maximal inhibitory concentration
IP: intraperitoneal injection
IV: intravenous injection
kDa: kilodalton
KD: dissociation constant
mAb: monoclonal antibody
MFI: mean fluorescence intensity
MGH: Massachusetts General Hospital
NHL: non‐Hodgkin's B‐cell lymphoma
Ni: nickel
nM: nanomolar
NOD/SCID: nonobese diabetic/severe combined immunodeficiency
*NSG*: NOD/SCID IL‐2 receptor γ^−/−^
PBMC: peripheral blood mononuclear cell
PE: phycoerythrin
SA: streptavidin
scFv: single‐chain variable fragment
SDS/PAGE: sodium dodecyl sulfate polyacrylamide gel electrophoresis
SD: standard deviation
VH: heavy chain variable domain
VL: light chain variable domain

## 1 Introduction

CD19 is a 95‐kDa transmembrane glycoprotein which is widely expressed on both normal and malignant B cells including chronic lymphatic lymphoma, non‐Hodgkin's B‐cell lymphoma, and B‐cell acute lymphoblastic leukemia (Turtle *et al*., 2016; Vallera *et al*., 2005). CD19 is known as a promising immunotherapy target. Anti‐human CD19 mAb drugs are already in the clinic or in clinical trials (Wang *et al*., 2012). However, effective antibody treatment requires the contributions of host effector mechanisms such as complement and NK cells. In contrast, immunotoxins are able to eliminate target cells even when host effector mechanisms are compromised due to chemotherapeutic agents or malnutrition. Some chemical‐conjugated anti‐human CD19 immunotoxins are already in clinical trial (Wang *et al*., 2012). However, these have varied degree of effectiveness and production difficulties (Vallera *et al*., 2005; Wayne *et al*., 2014). CD22‐CD19‐bispecific recombinant immunotoxin is in clinical trials (Bachanova *et al*., 2015; Vallera *et al*., 2005). However, its efficacy is dependent on target cells expressing CD22 and CD19 simultaneously. Previous attempts to produce a CD19‐specific recombinant immunotoxin have been unsuccessful (Du *et al*., 2008). We hypothesize that this was due to a suboptimal expression system. In this study, we developed a recombinant anti‐human CD19 immunotoxin using unique diphtheria toxin‐resistant yeast *Pichia Pastoris* expression system. The *in vitro* binding affinity and efficacy of the CD19 immunotoxin were assessed using human CD19^+^ mantle cell lymphoma cell line JeKo‐1. The *in vivo* efficacy of the CD19 immunotoxin was characterized using human CD19^+^ tumor‐bearing *NSG* mouse model.

## 2 Materials and methods

### 2.1 Cell lines and antibodies

Human CD19^+^ mantle cell lymphoma cell line JeKo‐1 (cat# CRL‐3006) and human CD19^−^ cell line CCRF‐CEM (cat# CCL‐119) were purchased from ATCC (Manassas, VA, USA). Human CD19^−^ cell line M14 and MD‐MBA‐231 were generously provided by Soldano Ferrone (Massachusetts General Hospital). mAbs used in this study are listed in Table 1.

**Table 1:** Antibodies used in this study

Antibody Name	Clone#	Source	Cat#
PE‐mouse anti‐human CD19	FMC63	Millipore	MAB1794H
APC‐mouse anti‐human CD4	L200	BD	551980
FITC‐mouse anti‐human CD3Ɛ	SP‐34‐2	BD	556611
PE‐mouse anti‐human CD3Ɛ	SP‐34‐2	BD	556612
PE‐mouse anti‐human CD19	HIB19	BioLegend	302208
APC‐mouse anti‐human CD8	RPA‐T8	BioLegend	301014
FITC‐anti‐human CD14	M5E2	BioLegend	301803
PE‐mouse anti‐human CD16	3G8	BioLegend	302008
PE‐mouse anti‐human CD194 (CCR4)	L291H4	BioLegend	359412
PE‐streptavidin		BioLegend	405204
FITC‐Annexin V		BioLegend	640906
7‐Aminoactinomycin (7‐AAD)		Sigma	A9400

### 2.2 DNA construction

**Figure 1:**
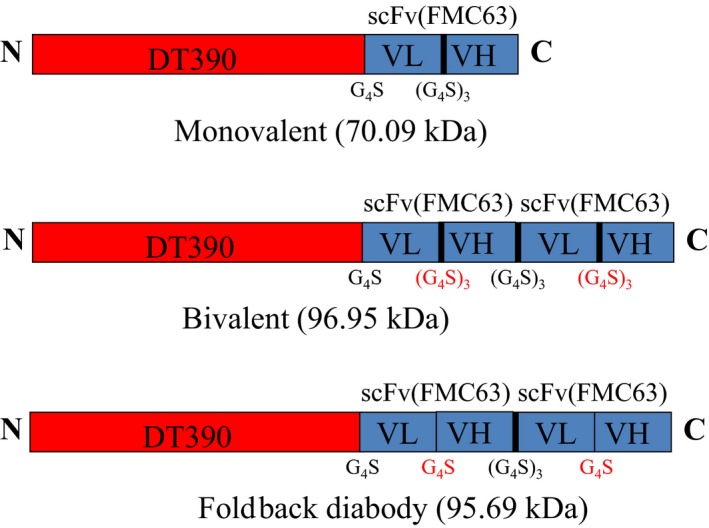
Schematic diagrams of the monovalent, bivalent, and single‐chain foldback diabody anti‐human CD19 immunotoxins.

**Figure 2:**
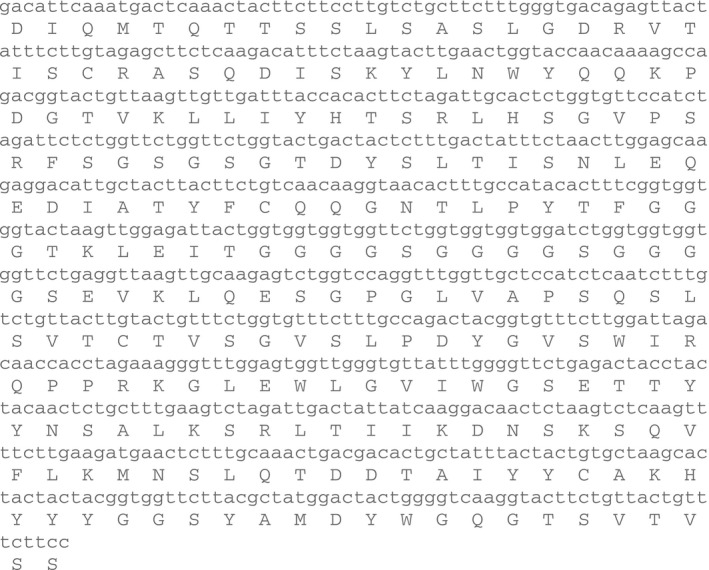
Codon‐optimized anti‐human CD19 scFv (FMC63) DNA and amino acid sequence.

As shown in Fig. 1, anti‐human CD19 immunotoxins were constructed using DT390 (Woo *et al*., 2002) and the codon‐optimized scFv (FMC63) nucleotide sequences (Figure 2, Nicholson *et al*., 1997). A strategy previously used to generate the anti‐human CCR4 immunotoxin (Wang *et al*., 2015) was applied to construct the anti‐human CD19 immunotoxins. DT390 and scFv (FMC63) or bi‐scFv (FMC63) are connected by a G_4_S (four glycines and one serine residue) linker. The two scFv (FMC63) of the bivalent immunotoxin are joined by (G_4_S)_3_ linker. Six histidines (6x His tag) were added to the C terminus of each construct to facilitate protein purification. To construct DT390‐scFv (FMC63), the codon‐optimized scFv (FMC63) was synthesized by GenScript and cloned into pwPICZalpha‐DT390 (Wang *et al*., 2011, 2015) between *Nco*I and *Eco*RI sites, yielding the final construct DT390‐scFv (FMC63) in pwPICZalpha. To construct DT390‐BiscFv (FMC63), the first scFv (FMC63) was amplified using PCR primers FMC‐Nco carrying *Xho*I and *Nco*I sites + FMC‐Bam1 carrying *Bam*HI and *Eco*RI sites and then cloned into pwPICZalpha between *Xho*I and *Eco*RI sites for sequencing confirmation. The insert was subsequently cut out with *Nco*I + *Bam*HI as insert I. The second scFv (FMC63) was PCR‐amplified using FMC‐Bam2 carrying *Xho*I and *Bam*HI sites + FMC‐Eco carrying an *Eco*RI site and then cloned into pwPICZalpha between *Xho*I and *Eco*RI sites for sequencing confirmation. The insert was then cut out with *Bam*HI + *Eco*RI as insert II. The insert I carrying *Nco*I and *Bam*HI sites + insert II carrying *Bam*HI and *Eco*RI sites [*Nco*I‐scFv (FMC63)‐*Bam*HI/*Bam*HI‐scFv (FMC63)‐*Eco*RI] were together cloned into pwPICZalpha‐DT390 between *Nco*I and *Eco*RI, yielding the final construct DT390‐BiscFv (FMC63) in pwPICZalpha. To construct the single‐chain foldback diabody CD19 immunotoxin, it is needed to build a modified scFv (FMC63) with a short linker (one G_4_S) between VL and VH. The VL portion was amplified using PCR primers FMC‐Nco + Bam‐FMC carrying *Bam*HI site and digested using *Bam*HI. The VH portion was amplified using PCR primers BgL‐FMC carrying *Bgl*II site + FMC‐Bam1 and digested using *Bgl* II. The *Bam*HI‐digested VL portion and *Bgl* II‐digested VH were ligated together for 4 h at room temperature as PCR template to amplify the scFv (FMC63) with short linker (one G_4_S) for constructing the single‐chain foldback diabody isoform following the construction procedure for DT390‐BiscFv (FMC63). All PCR primers that were used are listed in Table 2.

**Table 2:**
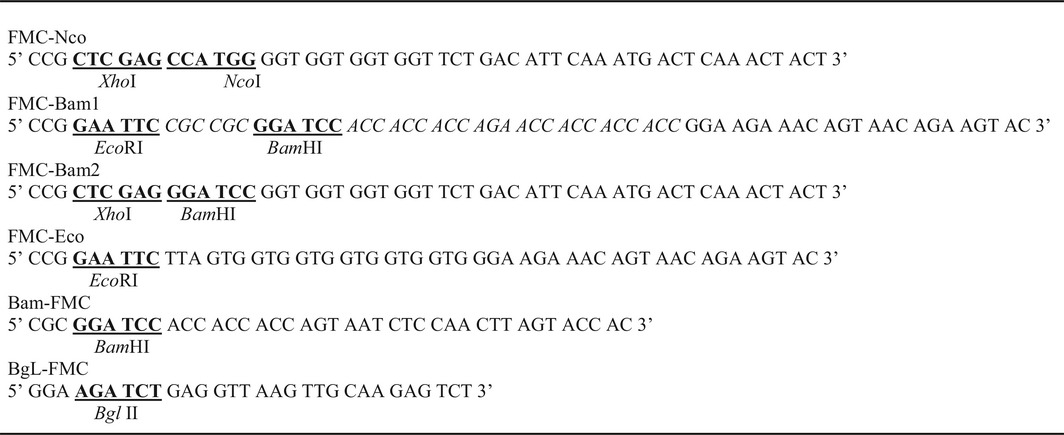
PCR primers used in this study

CD19 immunotoxin expression in the diphtheria toxin‐resistant yeast *Pichia Pastoris* expression system and purifications were performed as previously described (Peraino *et al*., 2013a; Wang *et al*., 2011). Western blot analysis, binding and blocking analysis by flow cytometry were all performed as previously described (Peraino *et al*., 2013a) using a human CD19^+^ mantle cell lymphoma cell line JeKo‐1. DT390 was used as negative control for *in vitro* functional analysis. C21 immunotoxin (a nonrelated DT390‐based immunotoxin) was used as negative control for *in vivo* efficacy characterization. Both DT390 and C21 immunotoxin were also expressed using yeast *Pichia Pastoris* expression system in our laboratory. Isolation of human PBMC, *in vitro* binding and depletion analysis of the CD19 immunotoxins to human blood, or PBMC with flow cytometry was performed as previously described (Wang *et al*., 2015).

### 2.3 *In vitro* efficacy analysis

*In vitro* efficacy of the CD19 immunotoxins was assessed using CellTiter‐Glo® Luminescent Cell Viability Assay (Promega, cat# G7571) to human CD19^+^ mantle cell lymphoma cell line JeKo‐1. Briefly, the JeKo‐1 cells were added to the wells of the opaque‐walled 96‐well plate at 10^4 ^cells/well. The CD19 immunotoxin was diluted in the tissue culture medium and added to the well at final concentration of 10^−6^, 10^−7^, 10^−8^, 10^−9^, 10^−10^, 10^−11^, and 10^−12^ m. The final volume is 100 μL per well for both JeKo‐1 cells and the CD19 immunotoxin. The control wells containing only the tissue culture medium were included to obtain the background luminescence value. Cycloheximide (1.25 mg·mL^−1^) was added to the positive control wells. The plates were incubated for 24 h at 37 °C, 5% CO_2_. The plates were equilibrated at room temperature for approximately 30 min. 100 μL of the CellTiter‐Glo® reagent was added and mixed for 2 min on an orbital shaker to induce cell lysis. The plate was then incubated at room temperature for 10 min to stabilize the luminescent signal, and the luminescence signals were recorded using Wallac Victor2 1420 multilabel counter (Perkin Elmer, Waltham, MA, USA).

### 2.4 *In vivo* efficacy analysis

The breeding pairs of *NSG* mice were purchased from Jackson laboratories (Bar Harbor, Maine) and bred in our rodent barrier facilities. All animal experiments were approved by Massachusetts General Hospital (MGH) Institutional Animal Care and Use Committee (IACUC). The *NSG* mice were divided into following groups: (a) C21 immunotoxin control group (a nonrelated diphtheria toxin‐based immunotoxin as negative control) (*n* = 7); (b) monovalent CD19 immunotoxin group (*n* = 7); (c) bivalent CD19 immunotoxin group (*n* = 7); and (d) single‐chain foldback diabody CD19 immunotoxin group (*n* = 7). All animals were injected IV with 10 million of human CD19^+^ mantle cell lymphoma cells (JeKo‐1) via the tail vein. The immunotoxin was injected IP from day 0 on at 100 μg·kg^−1^, BID for four consecutive days as one course, four courses in total, and three‐day break between any two courses. The injected animals were observed daily for signs and symptoms of illness and scored twice weekly based on the parameters as previously reported by our laboratory (Peraino *et al*., 2013b and Wang *et al*., 2015). In addition, to assess whether the CD19 immunotoxin is toxic to the animals, *NSG* mice (*n* = 2) injected with the bivalent CD19 immunotoxin only were also included as control.

### 2.5 Statistical analysis

All *p* values were calculated using log‐rank (Mantel–Cox) test of Prism. *p *<* *0.05 was considered as significant. IC_50_ was determined using nonlinear regression (curve fit) of Prism.

## 3 Results

### 3.1 Expression and purification of the CD19 immunotoxin

As shown in Fig. 1, monovalent, bivalent, and single‐chain foldback diabody versions of the anti‐human CD19 immunotoxins were constructed to find the isoform with the highest binding affinity and greatest ability to deplete human CD19^+^ cells. The codon‐optimized anti‐human CD19 scFv (FMC63) DNA (Fig. 2) (Nicholson *et al*., 1997) was cloned into the C terminus of the DT390‐containing yeast *Pichia Pastoris* expression vector pwPICZalpha‐DT390 between the *Nco*I and *Eco*RI sites (Wang *et al*., 2015). A 6xHis tag was added to the C terminus of each immunotoxin isoform to facilitate the purification. The DT390 domain was genetically linked to the scFv (FMC63) domain by a linker containing four glycine residues and a serine residue (G_4_S). For the monovalent and bivalent versions, the VL and VH were linked together by a long linker (G_4_S)_3_ to generate the scFv (FMC63). For the single‐chain foldback diabody isoform, the VL and VH were linked by a short linker G_4_S. The two scFv (FMC63) of the bivalent and the single‐chain foldback diabody isoforms were also joined by a long linker (G_4_S)_3_.

**Figure 3:**
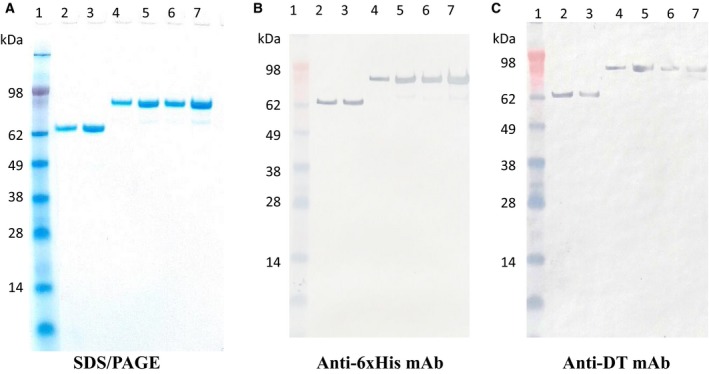
SDS/PAGE, western blot analysis of the anti‐human CD19 immunotoxins. (A) SDS/PAGE analysis (4–12% NuPAGE, Thermo Fisher Scientific); (B) western blot analysis using a mouse anti‐His mAb (clone#: 4A12E4, Thermo Fisher Scientific); (C) western blot analysis using a mouse antidiphtheria toxin mAb (clone# 3B6, Meridian). Lane 1: protein marker; Lanes 2–3: monovalent anti‐human CD19 immunotoxin [DT390‐scFv (FMC63), 70.09 kDa]; Lanes 4–5: bivalent anti‐human CD19 immunotoxin [DT390‐BiscFv (FMC63), 96.95 kDa]; Lanes 6–7: single‐chain foldback diabody anti‐human CD19 immunotoxin (95.69 kDa).

The CD19 immunotoxins were expressed using unique diphtheria toxin‐resistant yeast *Pichia Pastoris* (Liu *et al*., 2003) expression system in 1‐L Erlenmeyer flasks. The CD19 immunotoxins were captured using a Ni‐Sepharose fast flow resin and further purified using strong anion‐exchange resin. The final purification yield was ~ 15 mg per liter for the monovalent and ~ 6 mg per liter for the bivalent and ~ 2 mg per liter for the single‐chain foldback diabody CD19 immunotoxins of the original harvested supernatant. The purified anti‐human CD19 immunotoxins were analyzed by SDS/PAGE (Fig. 3A) and western blot using a mouse anti‐6xHis mAb (Fig. 3B) and a mouse antidiphtheria toxin mAb (Fig. 3C). SDS/PAGE and western blot analysis demonstrated that the three versions of the CD19 immunotoxins were expressed and purified with expected molecular weight of 70.09 kDa for the monovalent isoform, 96.95 kDa for the bivalent isoform, and 95.69 kDa for the single‐chain foldback diabody isoform.

### 3.2 Binding affinity analysis of the CD19 immunotoxins using flow cytometry

**Figure 4:**
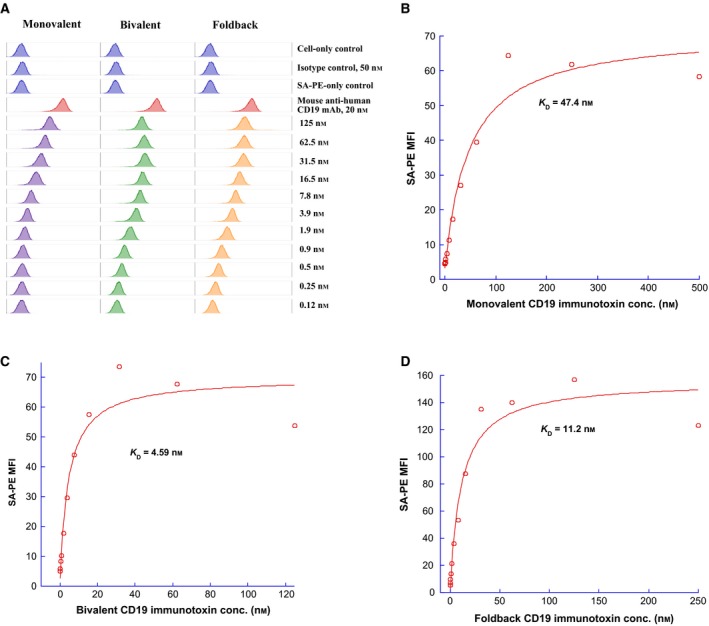
(A) Flow cytometry binding analysis of biotinylated monovalent anti‐human CD19 immunotoxin [DT390‐scFv (FMC63)] (left panel), bivalent anti‐human CD19 immunotoxin [DT390‐BiscFv (FMC63)] (middle panel), single‐chain foldback diabody anti‐human CD19 immunotoxin (right panel) to human CD19^+^ JeKo‐1 cells (mantle cell lymphoma cell line). Cells incubated with only the secondary staining (PE‐conjugated streptavidin) served as a negative control and PE‐mouse anti‐human CD19 mAb (clone#HIB19, BioLegend, cat#302208) for the positive control. PE‐mouse anti‐human CD194 (CCR4) (clone#L291H4, BioLegend, cat#359412) served as isotype control. Biotin‐labeled porcine CD3‐εγ (Peraino *et al*., 2012) was included as a negative control for background due to protein biotinylation. The data are representative of three individual experiments. (B‐D) *K*_D_ determination using flow cytometry and nonlinear least‐squares fit. MFI was plotted over a wide range of concentrations of biotinylated (B) DT390‐scFv (FMC63), (C) DT390‐BiscFv (FMC63), and (D) single‐chain foldback diabody anti‐human CD19 immunotoxin. The accompanying least‐squares fits are shown based on the hyperbolic equation y = m1 + m2 * m0/(m3 + m0), where y = MFI at the given biotinylated anti‐human CD19 immunotoxin concentration; m0 = biotinylated anti‐human CD19 immunotoxin concentration; m1 = MFI of zero biotinylated anti‐human CD19 immunotoxin control; m2 = MFI at saturation; and m3 = *K*_D_. A fitted *K*_D_ of 47.4 nm was obtained for DT390‐scFv (FMC63), 4.59 nm for DT390‐BiscFv (FMC63), and 11.2 nm for single‐chain foldback diabody anti‐human CD19 immunotoxin.

CD19 immunotoxins were designed to deplete human CD19^+^ cells via binding of the anti‐human CD19 scFv (FMC63) domain of the immunotoxins on the CD19 receptor of the CD19^+^ cells. Following the internalization, the released DT390 domain functions to inhibit protein synthesis resulting in the cell death (Murphy, 2011); therefore, the first step to characterize the CD19 immunotoxins was to analyze their binding affinity to human CD19 receptor. The anti‐human CD19 immunotoxins were biotin‐labeled for the binding analysis to human CD19^+^ JeKo‐1 cells using flow cytometry. As shown in Fig. 4A, monovalent (left panel), bivalent (middle panel), and single‐chain foldback diabody (right panel) CD19 immunotoxins bound to the human CD19 in a dose‐dependent manner. The binding affinity was quantified by the dissociation constant (*K*_*D*_) for each CD19 immunotoxin isoform using mean fluorescence intensity (MFI) (Peraino *et al*., 2013a). The foldback diabody isoform (*K*_D_ **= 11.22 nm, Fig. 4D) bound stronger than the monovalent isoform (*K*_D_ = 47.42 nm, Fig. 4B). However, the bivalent isoform exhibited the highest binding affinity (*K*_D_ = 4.59 nm, Fig. 4C).

**Figure 5:**
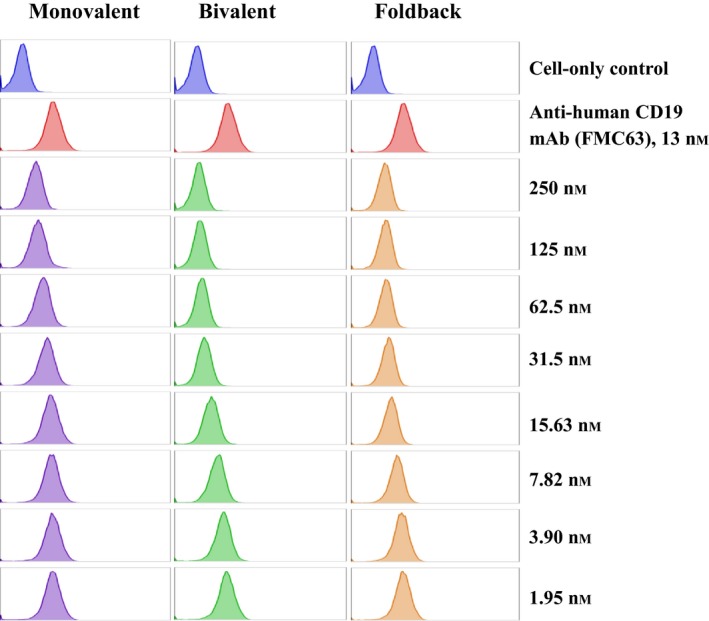
Blocking analysis of the anti‐human CD19 immunotoxins for the human CD19 receptor on human CD19^+^ JeKo‐1 lymphoma cells. Unlabeled anti‐human CD19 immunotoxins were each incubated with JeKo‐1 cells at a range of concentrations for 15 min at 4 °C in the dark. Subsequently, without washing the cells, anti‐human CD19 mAb (FMC63) was added to each tube containing cells in the presence of the unlabeled immunotoxins. Binding affinity of the anti‐human CD19 immunotoxins to the human CD19 receptor on JeKo‐1 cells was measured by a decrease in anti‐human CD19 mAb staining in the presence of increasing concentrations of the unlabeled immunotoxins.

The binding specificity of the CD19 immunotoxins was further analyzed by blocking the binding of the parent anti‐human CD19 mAb (FMC63) to its receptor on human CD19^+^ JeKo‐1 tumor cells. As shown in Fig. 5, monovalent (left panel), bivalent (middle panel), and single‐chain foldback diabody (right panel) CD19 immunotoxins blocked the binding of anti‐human CD19 mAb (FMC63) to the JeKo‐1 cells in a dose‐dependent fashion. The data strongly suggest that the CD19 immunotoxins bind specifically to the human CD19 receptor.

### 3.3 *In vitro* efficacy analysis of the CD19 immunotoxin

**Figure 6:**
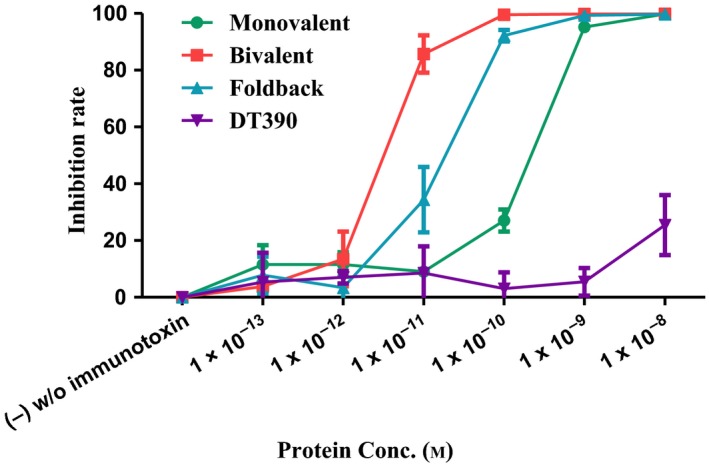
*In vitro* efficacy analysis of the CD19 immunotoxins using CellTiter‐Glo® Luminescent Cell Viability Assay (Promega, cat# G7571) to human CD19^+^ mantle cell lymphoma cell line JeKo‐1. (a) Monovalent anti‐human CD19 immunotoxin [DT390‐scFv (FMC63), green line]; (b) bivalent anti‐human CD19 immunotoxin [DT390‐BiscFv (FMC63), red line]; (c) single‐chain foldback diabody anti‐human CD19 immunotoxin (blue line); (d) DT390 alone (purple line). Y‐axis: inhibition rate of the cell viability by determining the number of viable cells based on the quantification of the ATP present. X‐axis: plated anti‐human CD19 immunotoxin concentration. Cycloheximide (1.25 mg·mL^−1^) was used as a positive control. The negative control contained cells without immunotoxin. *p *<* *0.0001 by log‐rank (Mantel–Cox) test of Prism (*n* = 3). Error bars indicate ±SD. Data are representative of multiple assays.

*In vitro* efficacy of the CD19 immunotoxins was assessed using CellTiter‐Glo® Luminescent Cell Viability Assay (Promega) in a human CD19^+^ JeKo‐1 tumor cell line. As shown in Fig. 6, all of the three CD19 immunotoxin isoforms (monovalent, bivalent, and foldback diabody) were effective. The foldback diabody isoform (IC_50_ = 1.7 × 10^−11^ m) was better than the monovalent isoform (IC_50_ = 2 × 10^−10^ m), while the bivalent isoform (IC_50_ = 2 × 10^−12^ m) was greatest. This findings correlate well with the corresponding binding affinity analysis.

### 3.4 *In vitro* binding and depletion analysis of the CD19 immunotoxins to human blood or PBMC

**Figure 7:**
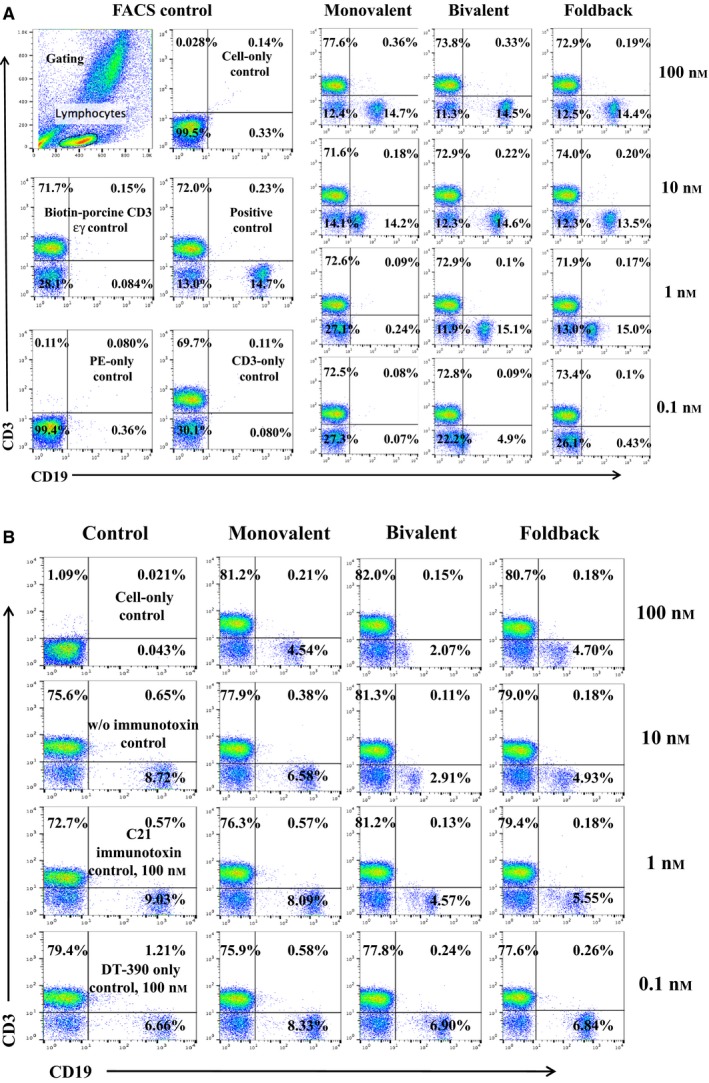
*In vitro* binding and depletion analysis of the anti‐human CD19 immunotoxins to human CD19^+^ blood or PBMC. (A) Flow cytometry binding analysis of the anti‐human CD19 immunotoxins to the CD19^+^ cells within human blood. Monovalent: monovalent anti‐human CD19 immunotoxin [DT390‐scFv (FMC63)]; bivalent: bivalent anti‐human CD19 immunotoxin [DT390‐BiscFv (FMC63)]; foldback: single‐chain foldback diabody anti‐human CD19 immunotoxin. PE‐SA: PE‐conjugated streptavidin; bio‐pCD3εγ: biotin‐labeled porcine CD3εγ (Peraino *et al*., 2012). (B) *In vitro* depletion of the CD19^+^ cells within human PBMC using the anti‐human CD19 immunotoxins. C21 immunotoxin and DT390 alone were included as negative controls. Data of both (A) and (B) are representative of three individual experiments.

CD19 is expressed on normal human B cells. To further characterize the CD19 immunotoxins, we performed *in vitro* binding and depletion analyses of the CD19 immunotoxins in human blood or PBMC. As shown in Fig. 7A, all three isoforms of the biotinylated CD19 immunotoxins bound to CD19^+^ human blood in a dose‐dependent fashion. The foldback diabody isoform bound more strongly than the monovalent isoform, while the bivalent isoform was best, which is consistent with the previous binding analysis using the human CD19^+^ JeKo‐1 cell line. We then further performed *in vitro* depletion analysis in CD19^+^ human PBMC using the CD19 immunotoxins. As shown in Fig. 7B, CD19^+^ human PBMC was depleted in a dose‐dependent manner following incubation of the CD19 immunotoxin with the human PBMC for 48 h. The bivalent and foldback diabody isoforms were better than the monovalent isoform, while the bivalent isoform was the most effective.

### 3.5 Off‐target analysis of the CD19 immunotoxin

**Figure 8:**
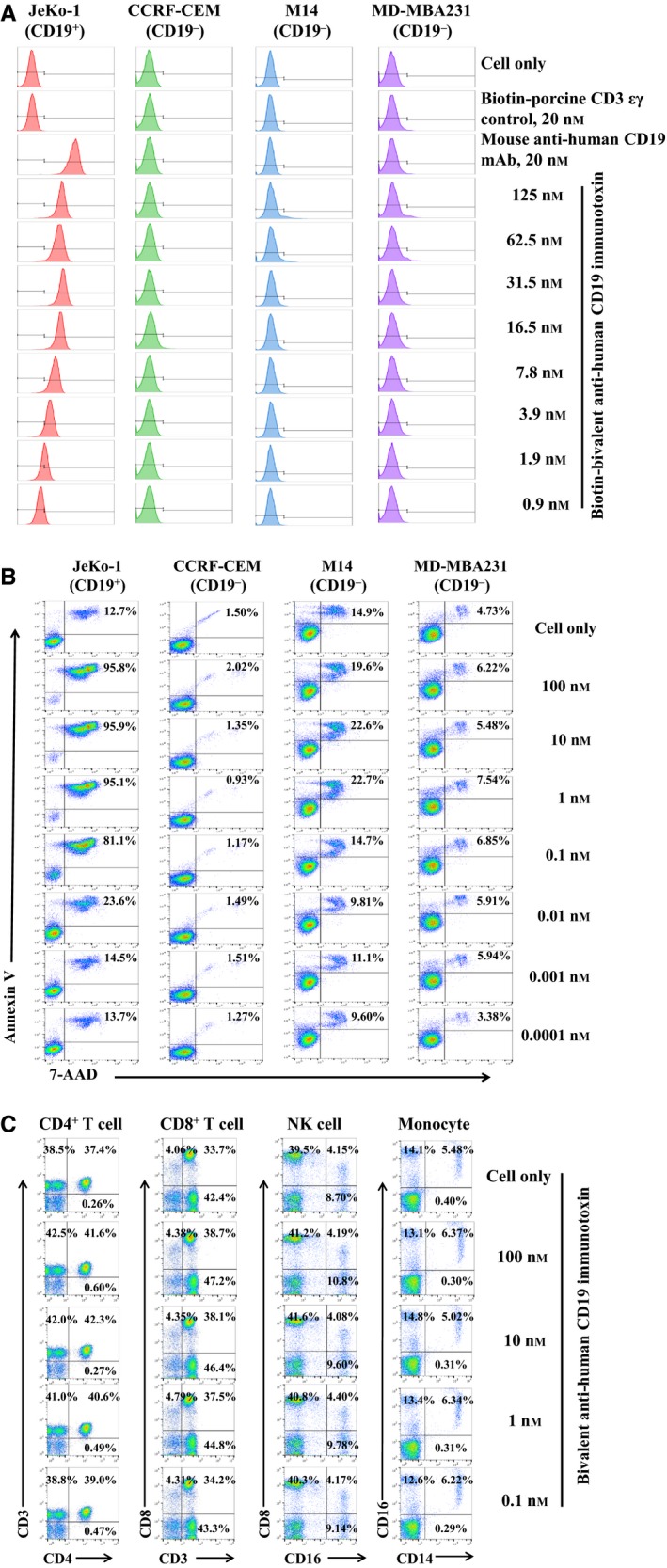
Off‐target analysis of the CD19 immunotoxin. (A) Flow cytometry binding analysis of the biotinylated bivalent anti‐human CD19 immunotoxin to three human CD19^−^ tumor cells (CCRF‐CEM, M14, and MD‐MBA‐231). PE‐mouse anti‐human CD19 mAb was included as positive antibody control, and biotinylated porcine CD3‐εγ (Peraino *et al*., 2012) as a negative control for background due to protein biotinylation. (B) Cell viability analysis of the three human CD19^−^ tumor cell lines by staining with 7‐ADD and Annexin V following incubation with the bivalent anti‐human CD19 immunotoxin for 18 h. (C) Depletion analysis by flow cytometry for the human CD19^−^ subpopulations within human PBMC following incubation with the bivalent anti‐human CD19 immunotoxin for 48 h. The data are representative of three individual experiments for all of the data in figure.

To rule out the off‐target effect of the CD19 immunotoxin, three human CD19^−^ tumor cell lines were analyzed with biotinylated bivalent human CD19 immunotoxin using flow cytometry: (a) CCRF‐CEM, (b) M14, and (c) MD‐MBA‐231. The results demonstrated that there was no binding activity with the three human CD19^−^ tumor cell lines (Fig. 8A). The viability of the three human CD19^−^ tumor cell lines was also analyzed by flow cytometry using 7‐aminoactinomycin D and Annexin V staining following incubation with the bivalent CD19 immunotoxin for 18 h. Their viability was not affected by this treatment (Fig. 8B).

To further rule out the off‐target effect of the CD19 immunotoxin, we also analyzed the human CD19^−^ PBMC populations following incubation with the bivalent CD19 immunotoxin for 48 h: CD4 T cell (CD3^+^CD4^+^), CD8 T cell (CD3^+^CD8^+^), NK cells (CD16^+^CD8^+^), and monocyte (CD14^+^CD16^+^). The results demonstrated that there was no effect on human CD19^−^ PBMC populations (Fig. 8C).

### 3.6 *In vivo* efficacy analysis of the CD19 immunotoxins

**Figure 9:**
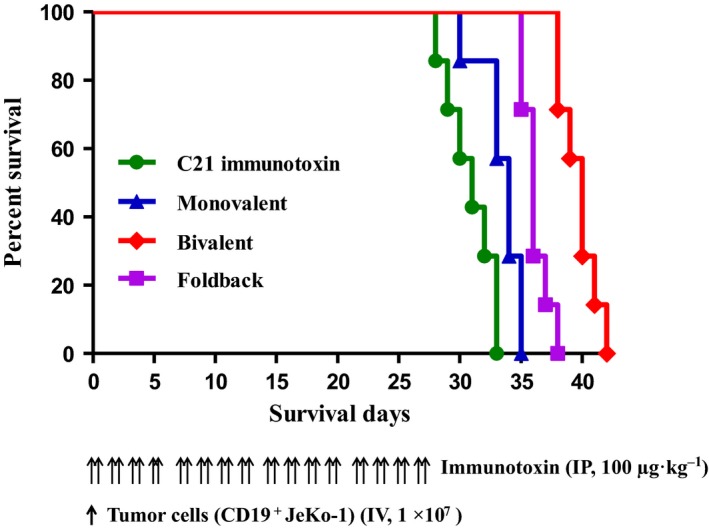
*In vivo* efficacy analysis of the anti‐human CD19 immunotoxins. *NSG* mice were injected IV with 1.0 × 10^7^ human CD19^+^ JeKo‐1 lymphoma cells and treated from day 0 on with the anti‐human CD19 immunotoxin (IP injection) at 100 μg·kg^−1^ BID for four consecutive days as one course, four courses in total, and three‐day break between any two courses. (a) C21 immunotoxin control group (a nonrelated DT390‐based immunotoxin as negative control) (*n* = 7, green curve) with a median survival time of 31 days; (b) monovalent anti‐human CD19 immunotoxin group (*n* = 7, blue curve) with a median survival time of 34 days; (c) bivalent anti‐human CD19 immunotoxin group (*n* = 7, red curve) with a median survival time of 40 days; (d) single‐chain foldback diabody anti‐human CD19 immunotoxin group (*n* = 7, purple curve) with a median survival time of 36 days. The schedule of drug and tumor cell injection is pictured in the schematic below the survival curve. The vertical arrows indicate the days on which the tumor cells or anti‐human CD19 immunotoxins were injected.

*In vivo* efficacy of the CD19 immunotoxins was assessed using human CD19^+^ JeKo‐1 mantle cell lymphoma‐bearing immunodeficient *NSG* mouse model. Human CD19^+^ JeKo‐1 cells (1 × 10^7^) were injected IV and the CD19 immunotoxins were injected IP at 100 μg·kg^−1^, BID, for four consecutive days as one course, four courses in total, and three‐day break between any two courses. As shown in Fig. 9, the C21 immunotoxin was included as a negative control (*n* = 7). These data demonstrated that all three CD19 immunotoxin isoforms prolonged animal survival significantly from a median of 31 days in the negative control group (C21 immunotoxin) to 34 days in the monovalent isoform treatment group (*n* = 7, *p *<* *0.0001), to 36 days in the foldback diabody isoform treatment group (*n* = 7, *p *<* *0.0001), to 40 days in the bivalent isoform treatment group (*n* = 7, *p *<* *0.0001) (Fig. 9). Mice receiving the CD19 immunotoxin only did not show any toxicity (data not shown). All animals injected with the human CD19^+^ JeKo‐1 cells succumbed to tumors (data not shown).

## 4 Discussion

Several attempts to develop recombinant anti‐human CD19 immunotoxins have been unsuccessful (Du *et al*., 2008). We speculate that the use of an inappropriate expression system was part of the problem. Diphtheria toxin‐resistant yeast *Pichia Pastoris* expression system is suitable for DT390‐based immunotoxin development. We speculate that eukaryotic expression systems such as yeast *Pichia Pastoris* expression system might be better than prokaryotic expression system such as *E. coli*.

Based on our previous experience, the foldback diabody immunotoxin isoform is superior to both bivalent and monovalent isoforms in terms of the *in vitro* binding affinity and efficacy (Kim *et al*., 2007; Wang *et al*., 2015). However, the foldback diabody isoform in this study did not improve the *in vitro* binding affinity and efficacy comparing with the bivalent isoform. Therefore, it is case by case for the immunotoxin development to find the best isoform (Wei *et al*., 2014).

The *in vivo* dosing schedule (Fig. 9) in this study has not been optimized. Our focus was simply to assess whether the CD19 immunotoxin functions *in vivo*. There is still room to increase the dose and duration to improve the animal survival. It is also necessary to characterize the *in vivo* efficacy further using established xenograft tumor‐bearing *NSG* mouse model. Furthermore, analyzing the residue tumor cells *in vitro* to determine whether they are still sensitive to the CD19 immunotoxin is warranted. If they remain sensitive, higher doses and multiple course of treatment could improve animal survival further.

Conventional chemotherapy and targeted mAb therapy have contributed significantly to the treatment for CD19^+^ B‐cell leukemia/lymphoma. However, there is still an unmet need to develop novel targeted therapies against the relapsing/refractory patients with CD19^+^ leukemia/lymphoma following the chemotherapy and targeted mAb therapy. We believe that CD19 immunotoxin will provide a novel alternative therapy for the refractory/relapsing CD19^+^ tumor patients.

## Author contributions

QZ and Zhaohui Wang primarily performed the experiments and data analysis as well as participated in writing the manuscript; HZ participated in the experiments; QH partially participated in the experiments; JCM, DHS, and CAH participated in the project design, data analysis, and writing the manuscript; Zhirui Wang primarily designed the project, analyzed the data, and wrote the manuscript.

## References

[mol212056-bib-0001] Bachanova V , Frankel AE , Cao Q , Lewis D , Grzywacz B , Verneris MR , Ustun C , Lazaryan A , McClune B , Warlick ED *et al* (2015) Phase I study of a bispecific ligand‐directed toxin targeting CD22 and CD19 (DT2219) for refractory B‐cell malignancies. Clin Cancer Res 21, 1267–1272. 25770294 10.1158/1078-0432.CCR-14-2877 PMC4360883

[mol212056-bib-0002] Du X , Beers R , Fitzgerald DJ and Pastan I (2008) Differential cellular internalization of anti‐CD19 and ‐CD22 immunotoxins results in different cytotoxic activity. Cancer Res 68, 6300–6305. 18676854 10.1158/0008-5472.CAN-08-0461 PMC2561922

[mol212056-bib-0003] Kim GB , Wang Z , Liu YY , Stavrou S , Mathias A , Goodwin KJ , Thomas JM and Neville DM Jr (2007) A fold‐back single‐chain diabody format enhances the bioactivity of an anti‐monkey anti‐CD3 recombinant diphtheria toxin based immunotoxin. Protein Eng Des Sel 20, 425–432. 17693455 10.1093/protein/gzm040

[mol212056-bib-0004] Liu YY , Woo JH and Neville DM Jr (2003) Targeted introduction of a diphtheria toxin resistant mutation into the chromosomal EF‐2 locus of *Pichia pastoris* and expression of immunotoxin in the EF‐2 mutants. Protein Expr Purif 30, 262. 12880776 10.1016/s1046-5928(03)00129-3

[mol212056-bib-0005] Murphy JR (2011) Mechanism of diphtheria toxin catalytic domain delivery to the eukaryotic cell cytosol and the cellular factors that directly participate in the process. Toxins 3, 294. 22069710 10.3390/toxins3030294 PMC3202816

[mol212056-bib-0006] Nicholson IC , Lenton KA , Little DJ , Decorso T , Lee FT , Scott AM , Zola H and Hohmann AW (1997) Construction and characterization of a functional CD19 specific single chain Fv fragment for immunotherapy of B lineage leukemia and lymphoma. Mol Immunol 34, 1157–1165. 9566763 10.1016/s0161-5890(97)00144-2

[mol212056-bib-0007] Peraino JS , Hermanrud CE , Springett L , Zhang H , Li G , Srinivasan S , Gusha A , Sachs DH , Huang CA and Wang Z (2012) Expression and characterization of recombinant soluble porcine CD3 ectodomain molecules: mapping the epitope of an anti‐porcine CD3 monoclonal antibody 898H2‐6‐15. Cell Immunol 276, 162. 22672968 10.1016/j.cellimm.2012.05.004 PMC3641788

[mol212056-bib-0008] Peraino JS , Schenk M , Li G , Zhang H , Farkash EA , Sachs DH , Huang CA , Duran‐Struuck R and Wang Z (2013b) Development of a diphtheria toxin‐based recombinant porcine IL‐2 fusion toxin for depleting porcine CD25^+ ^cells. J Immunol Methods 398–399, 33. 10.1016/j.jim.2013.09.006 PMC3840057 24055128

[mol212056-bib-0009] Peraino JS , Schenk M , Zhang H , Li G , Hermanrud CE , Neville DM Jr , Sachs DH , Huang CA , Duran‐Struuck R and Wang Z (2013a) A truncated diphtheria toxin based recombinant porcine CTLA‐4 fusion toxin. J Immunol Methods 391, 103. 23470981 10.1016/j.jim.2013.02.015 PMC3688055

[mol212056-bib-0010] Turtle CJ , Riddell SR and Maloney DG (2016) CD19‐Targeted chimeric antigen receptor‐modified T‐cell immunotherapy for B‐cell malignancies. Clin Pharmacol Ther 100, 252–258. 27170467 10.1002/cpt.392

[mol212056-bib-0011] Vallera DA , Todhunter DA , Kuroki DW , Shu Y , Sicheneder A and Chen H (2005) A bispecific recombinant immunotoxin, DT2219, targeting human CD19 and CD22 receptors in a mouse xenograft model of B‐cell leukemia/lymphoma. Clin Cancer Res 11, 3879–3888. 15897589 10.1158/1078-0432.CCR-04-2290

[mol212056-bib-0012] Wang Z , Duran‐Struuck R , Crepeau R , Matar A , Hanekamp I , Srinivasan S , Neville DM , Sachs DH and Huang CA (2011) Development of a diphtheria toxin based antiporcine CD3 recombinant immunotoxin. Bioconjug Chem 22, 2014–2020. 21866954 10.1021/bc200230h PMC3200572

[mol212056-bib-0013] Wang K , Wei G and Liu D (2012) CD19: a biomarker for B cell development, lymphoma diagnosis and therapy. Exp Hematol Oncol 1, 36. 23210908 10.1186/2162-3619-1-36 PMC3520838

[mol212056-bib-0014] Wang Z , Wei M , Zhang H , Chen H , Germana S , Huang CA , Madsen JC , Sachs DH and Wang Z (2015) Diphtheria‐toxin based anti‐human CCR4 immunotoxin for targeting human CCR4^+^ cells *in vivo* . Mol Oncol 9, 1458–1470. 25958791 10.1016/j.molonc.2015.04.004 PMC5528803

[mol212056-bib-0015] Wayne AS , Fitzgerald DJ , Kreitman RJ and Pastan I (2014) Immunotoxins for leukemia. Blood 123, 2470–2477. 24578503 10.1182/blood-2014-01-492256 PMC3990911

[mol212056-bib-0016] Wei M , Marino J , Trowell A , Zhang H , Peraino JS , Rajasekera PV , Madsen JC , Sachs DH , Huang CA , Benichou G *et al* (2014) Diphtheria toxin‐based recombinant murine IL‐2 fusion toxin for depleting murine regulatory T cells *in vivo* . Protein Eng Des Sel 27, 289–295. 25147093 10.1093/protein/gzu034

[mol212056-bib-0017] Woo JH , Liu YY , Mathias A , Stavrou S , Wang Z , Thompson J and Neville DM Jr (2002) Gene optimization is necessary to express a bivalent anti‐human anti‐T cell immunotoxin in *Pichia pastoris* . Protein Expr Purif 25, 270. 12135560 10.1016/s1046-5928(02)00009-8

